# Schwannoma of the lumbar spine, presenting with pain of the knee, like an osteoid osteoma, in a 10‐year‐old girl

**DOI:** 10.1002/ccr3.3503

**Published:** 2020-11-11

**Authors:** Nikolaos Laliotis, Chrysanthos Chrysanthou, Nikolaos Baskinis, Panagiotis Konstandinidis, Lambrini Giannakopoulou, Katerina Zarampouka

**Affiliations:** ^1^ Orthopaedic Department Interbalkan Medical Center Thessaloniki Greece; ^2^ Neurosurgical Department Interbalkan Medical Center Thessaloniki Greece; ^3^ Radiology Department Interbalkan Medical Center Thessaloniki Greece; ^4^ Pathology Department Interbalkan Medical Center Thessaloniki Greece

**Keywords:** child, pediatric tumor, referred pain, spinal schwannoma

## Abstract

Localized pain in the absence of local lesion may represent referred pain from the spine, in a child, arising from a benign spinal schwannoma. It can be diagnosed by MRI. Surgical excision of the tumor relieves the symptoms.

## Graphical abstract

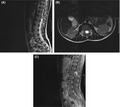

## 1 INTRODUCTION

A 10‐year‐old girl presented with severe pain of her knee, which had persisted over 6 months. Both radiological and clinical signs led to an incorrect diagnosis of osteoid osteoma. An MRI investigation of the spine confirmed the presence of spinal schwannoma. After surgical management, she had complete recovery.

Schwannomas are slow‐growing benign tumors arising from Schwann cells of the neural sheath. These tumors were previously described as neurilemmomas. Spinal schwannomas are solitary firm lobulated and encapsulated tumors arising mainly from the dorsal nerve root. In the spine, they can be located extradural, intradural extramedullary but rarely intramedullary. They are more common in the 4th to the 6th decade in life, being extremely rare in children. Their incidence is 0.3‐0.4 cases per 100 000 persons per year.^1^, ^2^, ^3^

Schwannomas are largely asymptomatic until they present symptoms. Pain and paresthesia are the main clinical symptoms. Motor disturbances, muscle weakness, or mass protruding are other rare clinical presentations.

We present a 10‐year‐old girl that had exacerbating pain only in the right knee, increasing during the night. An incorrect diagnosis of osteoid osteoma (OO) of the right knee was made, in accordance with the clinical symptoms. An MRI investigation confirmed the proper diagnosis of spinal schwannoma. The surgical treatment completely relieved her symptoms.

## 2 CLINICAL CASE PRESENTATION

A 10‐year‐old girl was referred to our pediatric orthopedic department for treatment with RF ablation of an osteoid osteoma of the right knee. Her past medical history was a 6‐month duration of severe pain, localized on the right knee, exacerbating during the night, making her unable to sleep. She was a normally developing girl, participating in sports activities until the appearance of pain. She had a series of blood test investigations (Hb, Ht, WBC, ESR, CRP, alkaline phosphatase) that were all in normal ranges.

**Figure 1:**
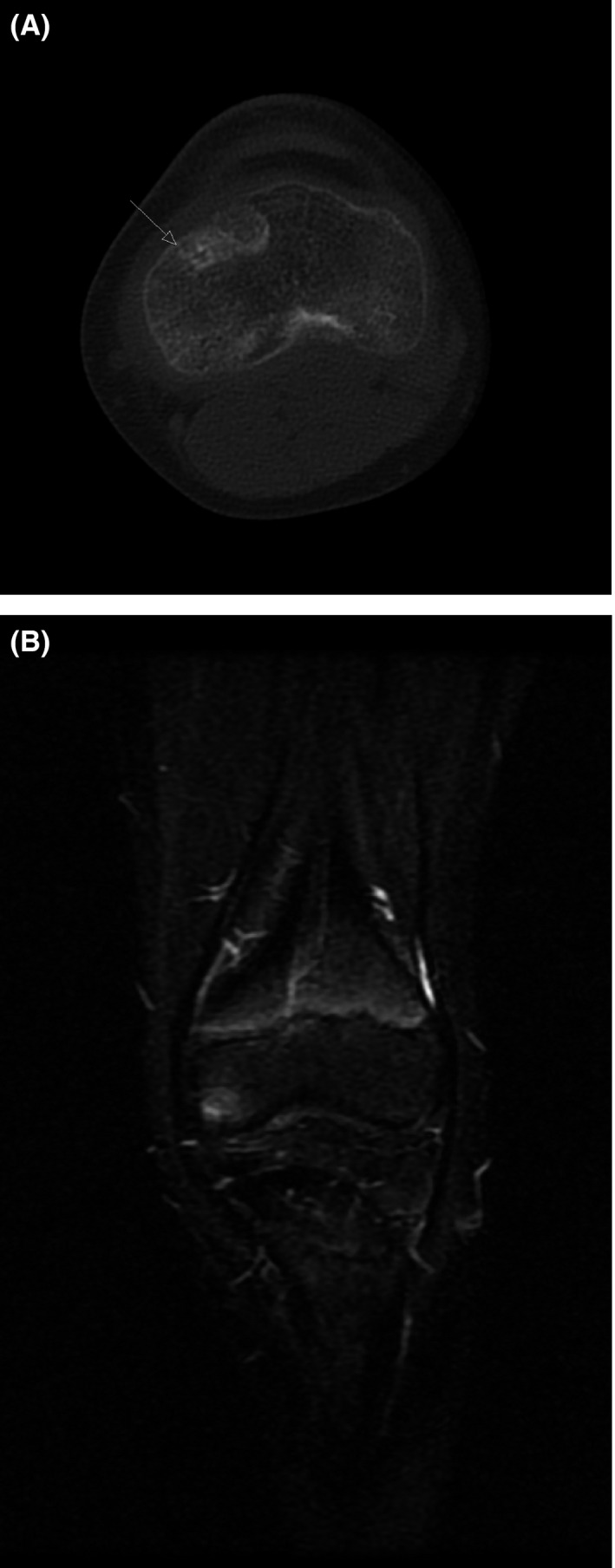
A‐B: Initial MRI and CT scan with an area of lucency with edema that led to incorrect diagnosis for a possible OO

Two X‐ray examinations of the right knee were taken in an attempt to define a diagnosis for the knee pain. An area of a small subperiosteal lucency of the lateral femoral condyle was reported. The right knee was further investigated with MRI. A consultant pediatric radiologist reported that there was minimal edema, possible small nidus, and absence of bone sclerosis. She suggested a diagnosis of a subchondral osteoid osteoma. The girl was provided with paracetamol and ibuprofen but the pain remained unchanged. Figure 1A‐B.

The girl was obviously suffering and was continuously grasping the right knee. She could walk normally without signs of limping and was able to hop bilaterally without discomfort. A clinical examination of the right knee revealed a full range of movements, without effusion. Both hips were also normal. Her spine was straight and with full movements of flexion and extension. She had normal bilateral tendon reflexes, a normal sensation in both of the legs and feet.

**Figure 2:**
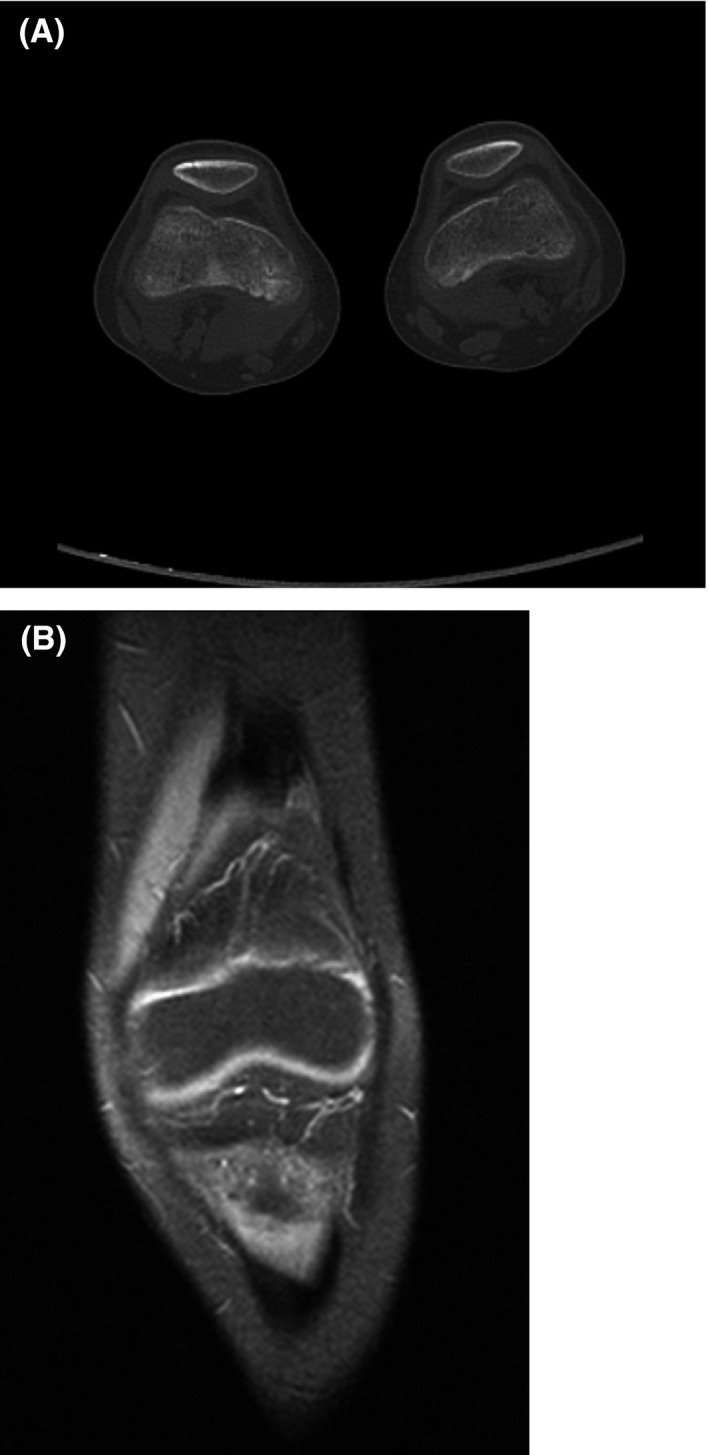
A‐B: Our CT and MRI examination with normal findings

Upon review of the X‐ray and MRI documents, we could not support the diagnosis of an osteoid osteoma (OO). Therefore, an MRI and a CT examination were undertaken with slices of 5 mm, but a nidus or sclerosis could not be identified. A bone scan was performed that had no increased uptake in the right knee. The bone scan had normal uptake throughout the body. We informed the patient's family that we would not precede to ablation. Figure 2A‐B.

We further examined her with an X‐ray of hips and pelvis, and lumbar spine AP and lateral, with normal findings. On the next day, we performed an MRI investigation of the right femur, pelvis, and lumbar spine in an attempt to find possible pathological signs.

**Figure 3:**
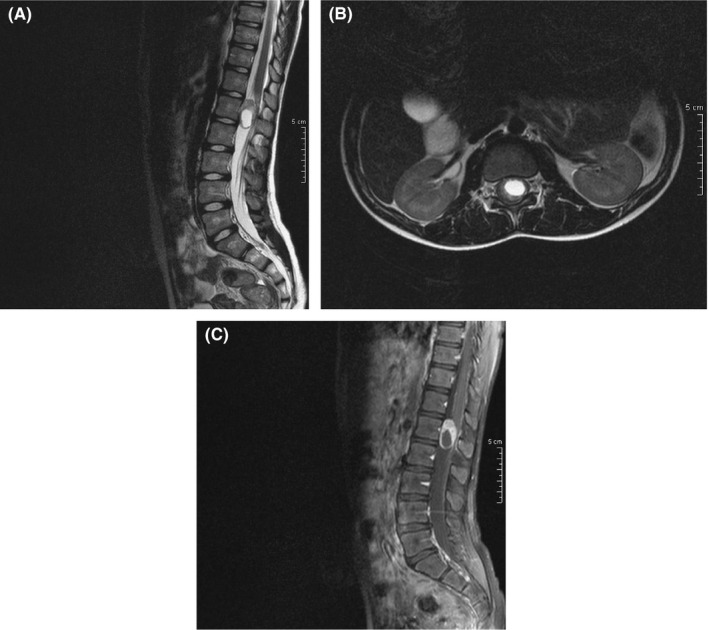
A, Sagittal T2W sequence. An oval intradural extramedullary lesion is observed at the level of the L1 vertebra. The central cystic part of the lesion demonstrates high signal in T2W sequences, whereas the peripheral solid part appears with intermediate signal. B, Axial T2W sequence. In the transverse plane, the lesion appears to be located in the central and left part of the thecal sac, causing pressure to the cauda equina. C, Sagittal T1W + C. After the intravenous administration of paramagnetic agent, there is vivid enhancement of the peripheral solid part of the tumor, with the central cystic component of the lesion remaining unenhanced

In the lumbar spine an oval, intradural extramedullary, tumefactive lesion was observed in the thecal sac, at the level of L1 vertebra. The lesion consisted of a cystic central part (showing a high signal in T2W and STIR sequences) and a solid peripheral part, with intense enhancement of the latter. There is no evident extension of the lesion in the foramina. The mass measured 27.5 mm (craniocaudal diameter) X 17.5 mm (transverse diameter) X 13 mm (anteroposterior diameter) Figure 3A‐C.

The lesion appeared to be mainly located in the central and left part of the thecal sac, causing pressure to the roots of cauda equina. The imaging findings were mostly consistent with a tumor of neurogenic origin (schwannoma). With the diagnosis of schwannoma of the spine, we informed the family about the need for surgical treatment of the tumor. We have not performed biopsy for a tumor with benign characters. Our neurosurgical team performed the operation. After posterior laminectomy of L_1_ and L_2_, a well‐defined capsulated tumor was resected after clear separation from the nerve roots.

**Figure 4:**
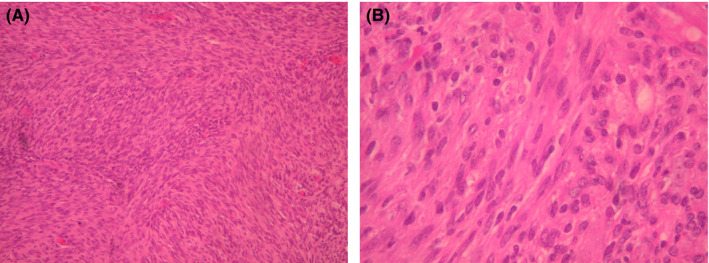
A, The tumor shows increased cellularity and the tumor cells are spindle‐shaped and arranged in fascicles. B, Tumor cells without nuclear atypia or mitosis

Histopathological and immunohistochemical analyses confirmed the diagnosis of a cellular neurilemmoma. The tumor showed increased cellularity and the cells were spindle‐shaped and arranged in fascicles. The tumor cells are without nuclear atypia or mitosis. Some show nuclear palisading. The tumor cells were strongly positive for S100 and negative for GFAP and EMA. The positivity for Ki67 in “hot spots” was about 5% of the nuclei. Figure 4A‐B.

The patient showed impressive recovery, feeling completely relieved. Peripheral neurological examination remained normal. We performed a new MRI of the spine in 6 months, with no elements of recurrence. The girl 8 months after the operation has not experienced any pain. The MRI examination of the brain was normal and we excluded neurofibromatosis.

## 3 DISCUSSION

Neurilemmomas or schwannomas are benign tumors that originate from Schwann cells. Spinal intradural extramedullary tumors are commonly meningiomas or schwannomas. Approximately 70% of spinal schwannomas arise from sensory roots, 20% from motor roots and 10% both from motor and sensory roots.^4^ Schwannomas are benign tumors that can grow considerably before producing symptoms. Sowash et al report on giant schwannomas that 20% were found incidentally.^5^

Handa et al report a series of 11 patients with giant sacral schwannomas in patients with mean age 53 years (35‐73) that had delayed diagnosis because it is a rare tumor with nonspecific symptoms.^6^ They authors stated that symptoms appear when the tumor expands in the sacral canal or destroys the bone. The classification of spinal schwannomas was described by Sridhar et al as types I‐V with subtypes, according to MRI findings. The types were based on the extension of the tumor into the canal and its extension into the foramen or on the erosion of the vertebral body.^7^

Osteoid osteomas are a common pathology in the 2nd decade of life, presenting with severe pain that occurs primarily at night. OOs mainly affect the long bones, producing typical radiological signs of bone sclerosis around an area of lucency with a central nidus. However, when OOs are located in the subchondral bone, there is minimal periosteum reaction and a diagnosis is usually difficult. MRI investigation of OO is mainly characterized by bone edema and is diffuse; it is not always accurate for obtaining a diagnosis of OO. In our patient, the initial examination was confused with normal subchondral nonossifying areas that are common findings without any clinical significance. But these areas were combined with severe knee pain at night, leading to a false diagnosis of OO. CT scan can provide an accurate localization of the nidus of an OO, assisting in the appropriate diagnosis. The bone scan is positive in active OO. We excluded the diagnosis of OO owing to the negative CT scan and bone scan.

The clinical presentation of schwannomas is mainly pain with neurological impairment according to the area that they originate. In a cohort of 831 patients (44.8 year old ± 13.2 years) with solitary schwannomas, pain was the main symptom (69.5%) followed by paresthesias and numbness (35.5%) and motor weakness (21.7%). Other symptoms such as difficulties in voiding, gait dizziness, fasciculation, and dysphagia were found in 4% of the patients.^8^ A schwannoma of the nerve root gave rise to extensive pain and numbness of the L_4_ distribution from torsion and hemorrhage in a healthy 62‐year‐old man.^9^ The pain was relieved after surgery.

Spinal schwannomas are extremely rare in children. Ustaris et al report a 4‐year‐old girl presented with toe walking pattern and progressive weakness of the lower legs, was diagnosed with a C_7_ schwannoma. Our patient had a normal peripheral neurological examination and showed no signs of nerve root impairment.^10^ Pokharel et al report an extradural cervical spinal schwannoma, in a 14‐year‐old child, that was presented with a single swelling of the posterior triangle of the neck with normal muscle strength and absence of sensory deficit.^4^ Yeh et al have published an 11‐year‐old boy with schwannoma of the C 6‐7 that was presented with forearm pain and progressive weakness and clawing hand.^11^ Mohanty et al reported another schwannoma of the neck in a 10‐year‐old boy, presented also with swelling in the neck and symptoms from swallowing and voice hoarseness that was arising from C_4_ body and with destruction of the vertebral body.^12^ Landi et al described the case of a 8‐year‐old female affected by a progressive paraparesis. She had a schwannoma at the level of T10‐T11.^13^

Our patient had only intense pain, without elements of radicular distribution and only her right knee was affected. There were no signs of radiculopathy or myelopathy, thus complicating the diagnosis. Clinical and radiological examination of the spine was normal. Pain is a particular feature in schwannomas of the cauda equine, caused both by compression of the sensory nerves and traction of the root.^14^ MRI is the method of choice for diagnosing soft tissue tumors of the spine. Plain radiographs and CT can reveal the osseous destruction caused by schwannomas. The two most common intradural extramedullary lesions of the lumbar spine are meningioma and schwannoma.

Spinal meningioma usually presents as a lesion iso‐ or hypointense to the spinal cord in T1W sequences and is mildly hyperintense to the cord in T2W sequences, with intense enhancement after the intravenous administration of paramagnetic agent. A characteristic dural tail sign may also be observed. Intraspinal schwannoma usually presents as a more heterogeneous lesion, which demonstrates low signal in T1W and predominately high signal in T2W sequences, with a high incidence of cystic areas centrally. The lesion also demonstrates intense enhancement after the intravenous administration of paramagnetic agent. In case the lesion extends into the neural foramen, the typical appearance of the “dumbbell sign” is observed, with widening of the foramen and vertebral scalloping. The lesion in our case was confined in the dural sac, with no foraminal extension.

In the differential diagnosis of intradural extramedullary lesions, other entities should also be included, such as myxopapillary ependymoma, paraganglioma, hemangiopericytoma, hemangioma, and metastases—which are more common in adults.^15^, ^16^

Surgical excision is the treatment of choice for spinal schwannomas. Complete resection of the tumor is recommended since partial resection has the risk of recurrence. When the tumor originates from motor roots there is a risk for motor deficit. Sridhar et al described surgical steps for giant erosive spinal schwannomas.^7^

It is important to extend our investigations, not only to the site of pain but to think also to referred pain from a spinal tumor.

## CONFLICT OF INTEREST

None declared.

## AUTHOR CONTRIBUTIONS

NL: is responsible for writing and editing the original draft. NB: performed surgery and is responsible for the investigation and collection of data. CC and KP: are responsible for the data collection and assisting in editing. LG: is responsible for radiological investigation. KZ: is responsible for the pathological examination.

## ETHICAL ISSUES

Full consent has been given by the parents for publication of the case report.

## Data Availability

There are no other data available.
